# Subjective Mood in Young Unmedicated Depressed Women under High and Low Sleep Pressure Conditions

**DOI:** 10.3390/biology5040052

**Published:** 2016-12-09

**Authors:** Angelina Birchler-Pedross, Sylvia Frey, Thomas Götz, Patrick Brunner, Vera Knoblauch, Anna Wirz-Justice, Sarah L. Chellappa, Christian Cajochen

**Affiliations:** 1 Centre for Chronobiology, Psychiatric Hospital of the University of Basel, Transfaculty Research Platform Molecular and Cognitive Neurosciences, University of Basel, Basel CH-4056, Switzerland; anbirchler@hotmail.com (A.B.-P.); syfrey@gmail.com (S.F.); dr.t.goetz@gmail.com (T.G.); patrick.brunner@usb.ch (P.B.); v.knoblauch@sleepmed.ch (V.K.); Anna.Wirz-Justice@unibas.ch (A.W.-J.); 2 Medical Chronobiology Program, Division of Sleep and Circadian Disorders, Brigham and Women’s Hospital; Division of Sleep Medicine, Harvard Medical School, Boston, MA 01215, USA; schellappa@partners.org

**Keywords:** major depressive disorder, sleep deprivation, mood, circadian rhythms, sleep homeostasis

## Abstract

Diurnal mood variations are one of the core symptoms in depression, and total sleep deprivation (SD) can induce rapid, short-lasting clinical improvement in depressed patients. Here, we investigated if differential sleep pressure conditions impact on subjective mood levels in young women with major depressive disorder (MDD) without sleep disturbances, and in healthy controls. Eight healthy and eight MDD women underwent 40-h SD (high sleep pressure) and 40-h multiple NAP (low sleep pressure) protocols under constant routine conditions during which subjective mood was assessed every 30-min. MDD women rated overall significantly worse mood than controls, with minimal values for both groups during the biological night (ca. 4 a.m.), under high and low sleep pressure conditions. During SD, nighttime mood ratings in MDD women were lower than in controls and partially recovered during the second day of SD, but never attained control levels. The degree of this diurnal time-course in mood under SD correlated positively with sleep quality in MDD women. Our data indicate that MDD women without sleep disturbances did not exhibit a SD-induced antidepressant response, suggesting that the mood enhancement response to sleep deprivation might be related to the co-existence of sleep disturbances, which is an association that remains to be fully established.

## 1. Introduction

Total sleep deprivation (SD) has been described as the most rapid non-pharmacological antidepressant currently known [1]. Indeed, marked improvements with SD may occur within hours in approximately 60% of depressive patients (reviewed in [1]). Even though the improvement following total SD is rapid, there is often a rapid return of depressive symptoms after subsequent recovery sleep, which suggests that the pathophysiological hallmarks of depression may be linked to sleep-wake regulatory processes [1]. Additionally, in order to optimize the antidepressant response, SD needs to coincide with an early morning circadian phase [2].

The theoretical framework for sleep deprivation as a means to improve subjective mood in depressive patients builds upon the two-process model [3,4] and its ramifications for depression [3,4]. Briefly, the process S-deficiency hypothesis postulates a deficiency in the homeostatic build-up of sleep pressure during wakefulness in people suffering with major depressive disorder (MDD), resulting in a shallower dissipation rate of process S during sleep [3,5]. Variations in homeostatic sleep regulation can be estimated by spectral electroencephalogram (EEG) activity in the low-frequency range (1–7 Hz) during both sleep and wakefulness [6,7]. Sleep in depression is often disturbed, as manifested by a reduction in slow-wave sleep (SWS) and a disinhibition of rapid eye movement sleep (REMS) [8,9]. The two-process model of sleep-wake regulation was proposed to explain sleep-wake cycle disturbances in major depression [3]. This model attempts to explain the effects of SD, REM-sleep disinhibition and reduction of SWS as the result of a deficient sleep homeostatic process that is linked with mood deterioration in depressive patients [3]. This putative relationship may not necessarily apply to all types of depression. Indeed, the process S-deficiency hypothesis for MDD has contradictory findings in sleep and wake EEGs. An early study observed lower delta waves during sleep in depressed patients without excluding for sleep disturbances [10]. In a similar vein, this finding was later observed in MDD individuals, albeit only in men [5]. Conversely, we previously showed that young MDD women devoid of co-morbid sleep disturbances exhibit higher, not lower, frontal non-REM delta EEG activity (0.75–4.5 Hz), a classical marker of homeostatic sleep pressure, during both baseline and recovery sleep subsequent to SD, as compared to that of healthy young and older women [11,12]. Furthermore, MDD women exhibited higher frontal low-frequency (FLA) wake EEG activity (0.5–5.0 Hz) during SD than that of healthy controls, particularly during the biological night [13]. Together, these data indicate that young, moderately depressed women without sleep disturbances do not exhibit a deficiency in the sleep homeostatic process S, as predicted by the S-deficiency hypothesis, but may rather live on an elevated level of homeostatic sleep pressure [11,12]. Thus, concurrent sleep disturbances may play a key role both in the pathophysiology of depression and in the antidepressant response to SD. Importantly, these findings may speak to the plethora of different depression subtypes and the underlying differences in sleep-wake regulation associated with these subtypes.

It still remains to be established if the antidepressant effects of SD are correlated with an amelioration of the proposed S-deficiency [3,4] and thereby with brief improvements in sleep quality, or if SD independently acts as an antidepressant. Thus, we investigated if differential sleep pressure levels (high vs. low), as a marker of the sleep homeostatic process, would have significant repercussions on self-rated mood in young women diagnosed with major depressive disorder (MDD) without sleep disturbances, as compared to age-matched healthy control women. Our main predictions were as follows:

1. Under high sleep pressure: MDD women without sleep disturbances will exhibit improvement in subjective mood during the course of a 40-h SD (high sleep pressure conditions), as compared to that of healthy control women. The SD-mood enhancement may occur due to the postulated deficiency in S-process in MDD individuals. Furthermore, the comparison to healthy age- and sex matched controls will allow us to tease apart how the SD-response is changed due to an underlying MDD (without co-morbid sleep disturbances) symptomatology.
2. Under low sleep pressure conditions: subjective mood ratings will not differ between MDD and control women during the course of a 40-h multiple NAP protocol, due to a minimal SD-response in both groups, and will undergo a pronounced circadian modulation.

## 2. Experimental Section

### 2.1. Study Participants

Control and MDD women were recruited via advertisement at different Swiss universities and through online job advertisement pages for students (for details see [13]). The rationale to include only women was due to the fact that, typically, women are twice as likely to experience MDD as compared to men. Considering that our study criteria included individuals that had MDD without any medication and without co-morbid sleep disturbances, it was extremely difficult to recruit men (the ratio of 2:1 women:men in MDD with medication and co-morbid sleep disturbances may be even more unbalanced given our study criteria) and to match both groups (MDD and controls) by age, sex, and body mass index (BMI). Thus, only women were included in the current study.

Our in-laboratory study enrolled 16 young MDD women (age range 20–31 years; age: 24 ± 4.8 years, mean ± standard deviation) who fulfilled the complete diagnostic criteria of the Diagnostic and Statistical Manual (DSM-IV) for MDD [14]. These participants were randomly allocated to either the low (*n* = 8) or high sleep pressure protocol (*n* = 8). This approach was used instead of a balanced crossover design to ensure the two-fold methodological aspects: (1) No experimentally-induced manipulation of prior sleep-wake history would potentially bias sleep-wake regulation and behavior (subjective mood) in all MDD participants; (2) To guarantee that, at the time of admission to the in-laboratory setting, all MDD participants had a stable clinical condition that would not be altered due to sleep-wake manipulations.

They had neither atypical symptoms nor other co-morbid psychiatric DSM-IV-disorders, and were without severe sleep problems as measured by the Pittsburgh Sleep Quality Index (PSQI ≤ 8) [15] (PSQI 5.5 ± 1.6). Each participant underwent a clinical interview with the same clinical psychologist (ABP). This interview was comprised of the structured clinical interview for DSM-IV Axis I Diagnoses of existing symptoms (SCID-I; 5.2 ± 0.4) [16]. The study volunteers had all of the following symptoms at the time of the SKID Interview: “sadness”, “diminished interest or pleasure”, “energy loss”, “reduced feeling of self-worth”, “diminished concentration”, and “social withdrawal”. Clinical status was further estimated by the Structured Interview Guide for the Hamilton Depression Rating Scale with Atypical Depression Supplement (SIGH-ADS) [17], which consists of the Hamilton-17 item scale (Standard Value ≥ 8; 12.39 ± 2.5) plus atypical items including the Montgomery-Åsberg Depression Scale [18] (MADRS: Standard Value ≥ 13; 16.7 ± 2.1) and the Beck Depression Inventory [19] (BDI: Standard Value ≥12); 21.3 ± 6.8). Two weeks after study completion, there was a follow-up assessment of the BDI (21.8 ± 9.1; *n* = 13), which confirmed the persistence of depressive symptoms.

The age-matched control group was comprised of eight healthy young women (age range 20–31 years; 25 ± 3.3 years) who were medically screened and had no prior psychiatric illness (for further detailed information on the recruitment of the control women see [20,21]).

None of the study participants were taking any medication, nor were they undergoing any type of medical or psychological treatment. All study participants were free of neurological and other sleep disorders, as assessed by a full-night polysomnography screening (PSG). To exclude chronotype-specific differences in circadian phase, only moderate chronotypes (morning-evening-type questionnaire rating between 14 and 21 points [22]) were selected. However, depressive study volunteers tended to be more evening types than healthy controls (16.1 ± 1.3 vs. 15.6 ± 3.7; *p* = 0.066, *t*-test). Body mass index (BMI) did not significantly differ between the groups (21.2 ± 2.5 vs. 20.9 ± 1.4 for the healthy volunteers). All participants were non-smokers, without any drug abuse, and were also required to abstain from heavy physical exercise and shift work before the study. All women started the study on days 1–5 after menses onset in order to complete the entire study block within the follicular phase, with the exception of three depressive and five control women who were taking oral contraceptives. The reason to control for menstrual phase is due to the influence of hormonal variations between follicular and luteal phases on readouts of the circadian timing system, including core body temperature and melatonin, and sleep-wake regulation [23]. The choice to include participants in the in-laboratory conditions during their follicular phase was due to the possible dampening of amplitude in circadian oscillation during the luteal phase [24]. Furthermore, increased daytime sleepiness in the luteal phase may be associated with increased daytime slow-wave sleep, plausibly through changes in thermoregulation during the luteal phase [24]. Although controversy still remains about the impact of different menstrual phases on outputs of sleep-wake regulation and the circadian system, accounting for these differences is crucial. Thus, to avoid a potential bias of different menstrual phases across participants impacting on key outputs of the circadian system, all participants started their in-laboratory conditions under the same menstrual (follicular) phase. All procedures conformed to the Declaration of Helsinki and the local Ethical Committee (Ethikkommission beider Basel, Basel, Switzerland). 

### 2.2. Protocol and Study Design

Each participant was instructed to maintain a regular sleep–wake cycle (sleep- and wake-times within ±30 min of self-selected target time), which was verified by wrist activity monitors (Cambridge Neurotechnology^®^, Cambridge, UK) and sleep logs during the one week prior to the study. None of our participants (healthy controls and MDD women) had sleep disturbances both subjectively and objectively prior to participating to the study. This was guaranteed by excluding any participants with a PSQI cut-off of 10 and above (which is deemed as clinically relevant poor sleep quality) and, most importantly, by having a sleep polysomnography night (adaptation night) prior to admission in the in-laboratory setting. Potential participant who had sleep efficiency below 80% and/or sleep disorders (i.e., sleep apnea, periodic limb movements, restless legs, and so forth) were excluded as study participants. The entire study design entailed two protocols: one for high and one for low sleep pressure conditions. The eight healthy control women underwent a crossover design thus participating in both protocols. In the depressive cohort, we recruited 16 study volunteers and distributed them randomly to either the high or low sleep pressure protocol. Each protocol was comprised of 3.5 days and started with an 8-h PSG night in the laboratory. During day 1, the study participants adjusted to the experimental dim light condition (<8 lux). After a second 8-h sleep episode, participants underwent either a high sleep pressure condition (40-h SD) or a low sleep pressure condition (40-h multiple NAP protocol) under strictly controlled constant routine (CR) conditions [7,25], which was followed by an 8-h recovery night sleep. The timing of the 8-h sleep episode was calculated with respect to the midpoint of each individual’s habitual sleep episode as assessed by actigraphy and sleep logs during the baseline week. All wake episodes were spent under semi-recumbent CR conditions during wakefulness and supine posture during scheduled sleep episodes.

### 2.3. Mood Ratings

During the study paradigm, the time-course of subjective mood was assessed in the MDD women by means of the Hamilton Depression Scale 7-Items (HAMD-7) German version analogous to [26] and the Visual Analogue Scale (VAS) for subjective mood. Subjective mood was assessed by a 100 mm bipolar visual analogue scale (VAS) at 30-min intervals in both MDD women and healthy controls. Participants were asked to indicate how they felt “at the moment” by placing a vertical mark on the VAS ranging from 0 (“worst ever”) to 100 mm (“best ever”). The VAS was administered every 30-min during scheduled wakefulness under a CR condition and every 30-min during the wake episodes under the NAP condition. To allow for a direct comparison between the two conditions, subjective mood ratings were collapsed into 3.75-h time bins per participant. Ultimately, this resulted in individual mood data every ca. 4 h during both in-laboratory conditions. The HAMD-7 which has been characterized for use in repeated short-term observer ratings, omitting sleep items that are not relevant during SD, was only assessed in the MDD women. The HAMD-7 encompassed the items “depressive mood”, “feeling of guilt”, “interest, pleasure, level of activity”, “tension and nervousness”, “physical symptoms of anxiety”, “energy level”, and “suicide”. A score ≥4 indicates that a given participant does not exhibit remission, while a score of ≤3 indicates full remission. This rating was carried out during the baseline week before the laboratory protocol started, and at four time points: at around 2 h (averaged time-of-day: 12 h) 14 h (21 h), 26 h (8 h), and 36 h (19 h) into the high or low sleep pressure protocol.

### 2.4. Data Analyses and Statistics

The statistical packages SAS^®^ (Version 6.12; SAS Institute Inc., Cary, NC, USA) and the SPSS^®^ (SPSS Inc., SPSS for Windows, Chicago, IL, USA, and Version 17) were used. For data reduction, subjective mood ratings were collapsed into 3.75-h time bins per participant before averaging over participants Individual 3.75-h averages of subjective mood-ratings entered a three-way repeated measure mixed-model analyses of variance (PROC MIXED, SAS) with main factors “group” (MDD women vs. healthy control women), sleep pressure condition (high vs. low), and “time-of-day” (11 time points). Since the three-way analyses on subjective mood did not yield significance for the interaction of “sleep pressure condition”, “time-of-day”, and “group”, we applied a data reduction approach, whereby two-way analyses with main factors “group” and “time-of-day” were performed for each protocol (SD and NAP) sleep pressure condition separately. All *p*-values were based on Huynh-Feldt’s (H-F) corrected degrees of freedom (significance level *p* < 0.05). Alpha adjustments for multiple comparisons were applied with the Tukey-Kramer test, which was also used for post-hoc comparisons. For the HAMD-7 observer ratings, a two-way repeated measure mixed-model analysis of variance (PROC MIXED, SAS) with main factors “condition” (high and low sleep pressure) and “time-of-day” (four time points) was applied. Lastly, diurnal changes in mood ratings were correlated (Pearson Product Moment correlation) with PSQI scores in MDD and control women during SD.

## 3. Results

### 3.1. Effect of Differential Sleep Pressure Conditions on Subjective Mood

**Figure 1:**
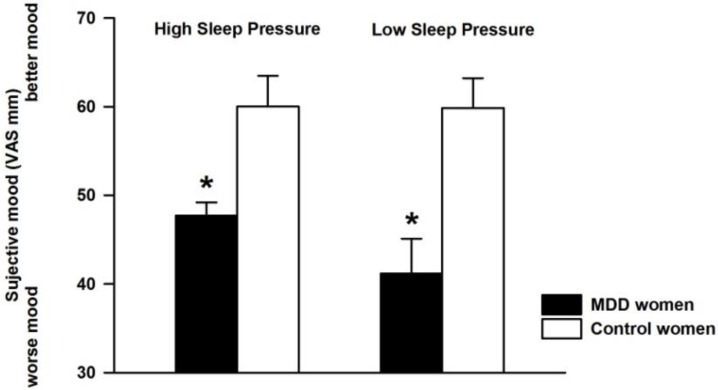
Subjective mood (Visual Analogue Scale—VAS) averaged for controls (white bars, *n* = 8) and major depressive disorder (MDD) women (black bars, *n* = 8) for high (left panel) and low sleep pressure (right panel). Data are presented as mean values ± standard error of the mean (SEM). * *p* < 0.05.

Average mood ratings across the low and high sleep pressure protocol were significantly lower in MDD than that of control women (Figure 1) (factor group: MDD women vs. healthy control; *F*_1,21.4_ = 17.88, *p* = 0.0004), whereas the factor “sleep pressure condition” and its interaction with the factor “group” both did not yield significance.

### 3.2. Time Course of Subjective Mood Levels under Different Sleep Pressure Conditions

**Figure 2:**
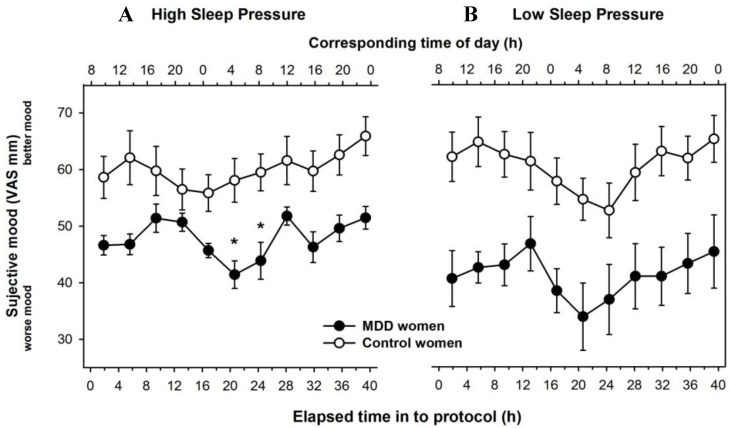
Time-course of subjective mood derived from the visual analogue scale during the 40-h high sleep pressure SD protocol (**A**) and during the 40-h low sleep pressure NAP protocol (**B**). Mean values ± SEM (*n* = 8 for controls and *n* = 16 for depressed, out of which *n* = 8 were assigned per sleep pressure condition). Data for MDD and control women are plotted in filled black and open symbols, respectively. Asterisks indicate significant post-hoc comparisons (Differences least squares mean values, *p* < 0.05).

The detailed time-course of mood ratings in 3.75-h time bins for MDD and control women is illustrated in Figure 2. Under high sleep pressure conditions, the time course of subjective mood significantly varied for the main factors “group” (MDD women vs. healthy control; *F*_1,19.7_ = 17.2; *p* = 0.0005), “time elapsed” (11 time points, *F*_10,136_ = 3.6; *p* = 0.0003), and the interaction of these two factors (*F*_10,136_ = 1.9; *p* = 0.04; Figure 2A). Post-hoc comparisons yielded significantly lower mood levels in MDD women during the second half of the night (4 a.m. and 8 a.m.).

Under low sleep pressure conditions, subjective mood yielded a significant group effect (*F*_1,18.9_ = 17.5; *p* = 0.0005), a tendency for a time effect (*F*_10,144_ = 1.8; *p* = 0.07), and no significant interaction of these effects (*F*_10,144_ = 0.7; *p* = n.s), (Figure 2B). MDD women rated their mood significantly worse irrespective of time-of-day in the NAP protocol, while both groups showed a distinct diurnal pattern with lowest mood ratings during the biological night, when endogenous melatonin values were highest.

### 3.3. Individual Time-Course of Subjective Mood

On a next step, we visually inspected individual time-courses for each participant (young controls and MDD women). Under SD, 7/8 young controls had overall high mood levels (above cut-off of 50 in the VAS) over 40 h and the single participant with lower mood levels had an average of around 45 (Figure S1A). Conversely, 7/8 MDD participants had low mood self-ratings (<50 in the VAS) at virtually all time-points (Figure S1C). The single participant who had higher mood levels was not a statistical outlier (within ±1 standard deviation from the group average). Under low sleep pressure conditions, 7/8 young controls had high mood levels (Figure S1B), whereas all MDD women had low mood ratings across the entire study paradigm (Figure S1D). None of the MDD women had better mood on day 2 than on day 1 of the SD protocol, thus no antidepressant effect was induced.

### 3.4. HAMD-7 Observer Ratings in MDD Women

The time-course analysis of VAS mood self-ratings was complemented by analysis of the observer ratings derived from the HAMD-7 questionnaire, which indices core symptoms of depression. A tendency for significance was observed for the main effect “condition” (*F*_3,26_ = 1.1; *p* = 0.08), such that under low sleep pressure, MDD women had fewer depression symptoms than under SD (Figure S2). There were no significant differences for the main effect “time-of-day” and the interaction of “condition” and “time-of-day”.

### 3.5. Sleep Deprivation Effect and Severity of PSQI Value in MDD Women

**Figure 3:**
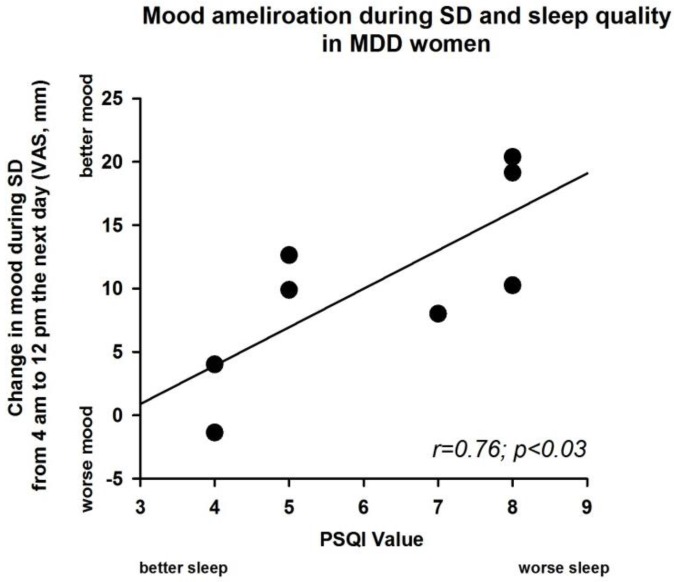
Linear regression between the values of the Pittsburgh Sleep Quality Index (PSQI) at baseline and the values of mood change from the visual analogue scale in the high sleep pressure protocol for MDD women (*r* = 0.76; *p* < 0.03). This figure shows the difference of subjective mood between clock time 4 a.m. (20-h after elapsed time awake) and clock time 12 p.m. (28-h after elapsed time awake) (see Figure 2, high sleep pressure protocol). *X*-axis: PSQI values; *Y*-axis, VAS of subjective mood. Black dots correspond to individual participants.

Given the significant interaction between factors “group” and “time” and the visual inspection of the individual mood curves during SD, we defined an index of “diurnality” by calculating the night-to daytime difference in mood ratings for each individual. To this end, we subtracted the night-time rating at 4 a.m. (20-h after elapsed time awake) from the daytime rating at noon the next day (12 p.m.; 28-h after elapsed time awake) during high sleep pressure conditions (i.e., SD). A linear regression between the PSQI-values (indicator of sleep quality) and this diurnality index in subjective mood yielded a significant *r*-value (*r* = 0.76; *p* < 0.03 (Figure 3) in MDD women but not in control women (*r* = −0.47, n.s).

## 4. Discussion

Our data do not support the two postulated hypotheses. First, in young, unmedicated women with MDD and no sleep disturbances, total SD did not lead to a significant antidepressant effect, when comparing subjective mood ratings (derived from a visual analogue scale) or the HAMD-7 items observer rating from day 1 with those from day 2. Both scales documented a similar time course, important for the validity of our findings. These results were further confirmed by the individual time-courses, such that no MDD participants exhibited SD-related mood enhancement. Collectively, the data suggest that SD yields no antidepressant effect in young depressive women with major depression without concomitant sleep disturbances. Second, under the low sleep pressure conditions of a NAP protocol, MDD women still had lower subjective mood ratings than control women. All groups showed circadian modulation of mood independent of the sleep pressure condition.

The lack of SD-mood enhancement found may be related to the selected group of young depressed women without sleep complaints. Our study sample was diagnosed according to DSM-IV [14] with MDD without any sleep complaints, in order to study the underlying depressive pathophysiology not related to sleep disorders (additionally, for this reason, participants were unmedicated). The findings suggest that there may be differences in the mood-enhancing response to SD between MDD with and without sleep disturbances.

Some studies have provided insights into sleep-related changes associated to MDD. The distribution of slow-wave activity (SWA), as indexed by the delta sleep ratio [quotient of SWA in the first to the second non-Rapid eye movement sleep (NREM) episode], was investigated in unmedicated SD responders and SD non-responders [27], whereby a high delta sleep ratio was a positive predictor for SD response. We previously described that the same MDD women as in this analysis appear to live on a higher level of homeostatic sleep pressure, as indexed by NREM EEG slow-wave activity (SWA 0.75–4.5 Hz) during baseline sleep [11,12]. Recovery sleep at the end of the high and also low sleep pressure protocols also showed significantly higher SWA in the MDD women than in controls [11,12]. Moreover, MDD women responded with a stronger EEG synchronization in a frequency range of 0.5–5 Hz during SD [13], which confirmed the higher delta waves during sleep, but not the role of delta sleep as a positive predictor for the SD response. These results challenge a generalized assumption that “Process S” is deficient in all MDD [3]. Conversely, a higher level of sleep pressure may be a negative predictor of response to sleep deprivation. SD response may depend on the degree of concomitant sleep disorder. Indeed, within the small group undergoing SD, worse sleep in the PSQI correlated with a greater improvement on day 2.

If MDD women with no sleep disturbances appear to live under higher sleep pressure, one may argue this “ceiling effect” does not allow for further increases in sleep pressure that would be triggered by SD. Ultimately, this may account for the absence of SD-antidepressant effects. Interestingly, selectively reducing slow-waves during sleep with low level acoustic stimuli (slow-wave deprivation; SWD), without disrupting total sleep time, has been shown to acutely reduce moderate MDD symptomatology [28]. Participants experienced a significant decrease in depressive symptoms according to both self-rated and researcher-administered scales, which was correlated with the overnight dissipation of frontal-central slow-wave activity (SWA) during baseline sleep and the rebound in right frontal all-night SWA during recovery sleep [28]. Given these data, one could speculate that MDD—when dissociated from co-morbid sleep disturbances—may be associated with an overall higher sleep pressure, as compared to that of MDD associated with insomnia.

Insomnia complaints tend to precede the onset and recurrence of depression [29,30,31] in as many as 40% of cases [32]. The risk of developing major depression is significantly increased in individuals suffering from insomnia [33,34,35]. An important corollary is that subjectively reported better sleep quality post-treatment is associated with lower rates of depression recurrence [36]. Apart from sleep-wake impact on mood, the circadian timing system is a known key player on mood regulation in both healthy [21] and depressed individuals [37]. Thus, both the duration of wakefulness and the circadian pacemaker are likely involved in the regulation and dysregulation of mood [37]. Under low sleep pressure conditions, the time-course across all MDD women (Figure 2) and their individual data (Figure S1) both indicate lower mood variability over time, which may reflect a flatted circadian variation of mood. Together, these observations suggest a critical role played by sleep-wake regulatory and circadian mechanisms in the pathophysiology of depression.

Limitations to our current findings include the relatively low sample size, which warrant replication in larger study cohorts, and the absence of a separate MDD cohort with insomnia, which is the sleep disorder most frequently co-morbid to this depression subtype. By experimentally manipulating prior sleep-wake history in this potential group, one might tease apart the potential role that insomnia would have in SD-mood responses. Future studies are warranted to specifically investigate this association on subjective mood in MDD individuals.

## 5. Conclusions

Our data indicate that MDD women without sleep disturbances did not exhibit an SD-induced antidepressant response, suggesting that the mood enhancement response to sleep deprivation might be related to the co-existence of sleep disturbances. Future studies are warranted to establish this association in patients suffering from MDD but without concomitant sleep disturbances.

## Supplementary Materials

The following are available online at www.mdpi.com/2079-7737/5/4/52/s1, Figure S1: Individual time-course profiles of subjective mood, Figure S2: Time course of the Hamilton Depression Rating Scale in MDD women.
