# Supplementary material for: Subjective Mood in Young Unmedicated Depressed Women under High and Low Sleep Pressure Conditions
Source: Biology (Basel). 2016 Dec 9;5(4):52. doi: 10.3390/biology5040052 (PMC5192432; doi:10.3390/biology5040052)
Supplement: Supplementary file 1 [file biology-05-00052-s001.docx]

Supplementary Materials: Subjective Mood in Young Unmedicated Depressed Women under High and Low Sleep Pressure Conditions

Angelina Birchler-Pedross, Sylvia Frey, Thomas Götz, Patrick Brunner, Vera Knoblauch,
Anna Wirz-Justice, Sarah L. Chellappa and Christian Cajochen

**Figure S1.** Individual time-course profiles of subjective mood (from the visual analogue scale) in young controls (A–B) and in major depressive disorder (MDD) women (C–D) during a 40-h high sleep pressure protocol (A,C; solid lines represent each individual time-course, eight young controls and eight MDD women) and during a 40-h low sleep pressure protocol (B,D; dashed lines represent each individual time-course, eight young controls and eight MDD women). Identical colors label the same individual under both protocols.





**Figure S2.** Time course of the Hamilton Depression Rating Scale (HAMD-7) items observer ratings in MDD women during the high (filled triangle line) and low (open triangle line) sleep pressure protocol. Mean values ± SEM (*n* = 8, different subjects per sleep condition).
